# Development and Validation of Gas Chromatography-Triple Quadrupole Mass Spectrometric Method for Quantitative Determination of Regulated Plasticizers in Medical Infusion Sets

**DOI:** 10.1155/2018/9470254

**Published:** 2018-02-05

**Authors:** So Hyeon Jeon, Yong Pyo Kim, Younglim Kho, Jeoung Hwa Shin, Won Hyun Ji, Yun Gyong Ahn

**Affiliations:** ^1^Western Seoul Center, Korea Basic Science Institute, Seoul 03759, Republic of Korea; ^2^Department of Environmental Science and Engineering, Ewha Womans University, Seoul 03759, Republic of Korea; ^3^Department of Chemical Engineering and Material Science, Ewha Womans University, Seoul 03760, Republic of Korea; ^4^Department of Health, Environment & Safety, Eulji University, Seongnam 13135, Republic of Korea; ^5^Seoul Center, Korea Basic Science Institute, Seoul 02841, Republic of Korea; ^6^Institute of Mine Reclamation Technology, Mine Reclamation Corporation, Wonju 26464, Republic of Korea

## Abstract

A method for the quantitative determination of dibutyl phthalate (DBP), benzyl butyl phthalate (BBP), bis(2-ethylhexyl) adipate (DEHA), bis(2-ethylhexyl) phthalate (DEHP), di-n-octyl phthalate (DNOP), dioctyl terephthalate (DOTP), diisononyl phthalate (DINP), and diisodecyl phthalate (DIDP) in medical infusion sets was developed and validated using gas chromatography coupled with triple quadrupole mass spectrometry (GC-MS/MS) in the multiple reaction monitoring (MRM) mode. Solvent extraction with polymer dissolution for sample preparation was employed prior to GC-MS/MS analysis. Average recoveries of the eight target analytes are typically in the range of 91.8–122% with the relative standard deviations of 1.8–17.8%. The limits of quantification (LOQs) of the analytical method were in the ranges of 54.1 to 76.3 ng/g. Analysis using GC-MS/MS provided reliable performance, as well as higher sensitivity and selectivity than GC-MS analysis, especially for the presence of minority plasticizers at different concentrations.

## 1. Introduction

Phthalates as a class of synthetic chemicals are widely used in a variety of consumer products including medical devices, toys for children, food wrapper, building materials, automotive parts, and so on [1–3]. Since they may cause harm to human health by altering endocrine function or through other biological mechanisms [4], environmental monitoring of phthalates has been accomplished from various media (air, soil, food, water, etc.) [5–9]. Most medical devices are made of flexible polyvinylchloride (PVC), a produced synthetic plastic polymer due to its numerous benefits, which include chemical stability, biocompatibility, clarity and transparency, flexibility, durability, chemical and mechanical resistance, sterilizability, and low cost [10]. Since plastics are mainly used as plasticizers to soften PVC, phthalates are abundant in PVC-based medical devices, and they may enter into contact with the patients through leaching out into infused solutions [11, 12]. The larger molecular weight phthalates—di(2-ethylhexyl) phthalate (DEHP), di-n-butyl phthalate (DBP), and diisononyl phthalate (DINP)—are suspected carcinogens, toxic to liver, kidneys [13], and reproductive organs [14]. Benzyl butyl phthalate (BBP), DBP, and DEHP are weakly estrogenic [15]. There have been reports of extraction of phthalates such as BBP, DBP, and DEHP in dialysis tubing and infusion bags [16, 17]. Consequently, the European Chemicals Agency (ECHA) recommended that several compounds of very high concern should not be used without specific authorization. Furthermore, according to South Korea Ministry of Food and Drug Safety (MFDS) regulation, phthalates within intravascular administration products including DEHP, DBP, and BBP are no longer being used in the production of medical infusion sets since July 2015.

Due to the expanding use of phthalates and assimilated analytes in medical devices, the analytical methods for plasticizers from infusion sets are increasing in order to create plans for safety. Several analytical methods capable of detecting and quantifying the alternative plasticizers in medical devices have been developed and validated by means of separative and nonseparative methods up to date [18]. The separative methods have been adopted with gas chromatography, supercritical fluid chromatography [19], and liquid chromatography combined with various detectors such as mass spectrometer, flame ionization detector, evaporative light scattering detector, or UV techniques. Nonseparative methods have been performed on a nuclear magnetic resonance and Fourier transform infrared spectrometry [20, 21]. Among these methods, gas chromatography coupled with mass spectrometry was the most specific and sensitive method and suggested to be a suitable method to perform a regulatory control. Furthermore, application of GC-triple quadrupole mass spectrometry (GC-MS/MS) is recently increasing in terms of quantitative confirmation in various matrices [22].

For the sample preparation, an easy and inexpensive technique is polymer dissolution which is well known as a solid-liquid extraction [23]. The whole polymer is first dissolved in a solvent-like tetrahydrofuran (THF) or dimethylacetamide [24, 25]. After then, when solvent is added to the dissolution for solvent extraction, PVC-based polymers are removed by precipitation. There are already two publications by Gimeno et al. and Bourdeaux et al. dealing with the GC-MS analysis of phthalates and assimilated analytes in medical devices [26, 27]. But the viewpoint of the detection level and target analytes is clearly different from our study. It is important to monitor the presence of the minority plasticizers, as well as the majority, because their migration and toxic potential can be extremely different depending on their chemical properties. Furthermore, alternative plasticizers and mixtures have been increasingly developed to provide flexibility for medical devices in these days. Also, their concentration levels in the samples can be quite varied, but they should be evaluated depending on the type of phthalates and assimilated analytes. Accordingly, the identification and quantification of toxic plasticizers in medical devices, especially the tubings of medical infusion sets, are needed since they are able to leach from infusion sets and result in human exposure directly.

The aim of this study was to evaluate the capabilities of GC-MS/MS for the quantification of phthalates and assimilated analytes from infusion sets. Analysis using GC-MS with a single quadrupole mass analyzer has been typically used in most cases for the determination of phthalates and assimilated analytes in medical devices. However, this method requires a clean matrix to avoid the interference of unwanted ions [28]. By conducting interlaboratory collaborative studies, it was found that there was a lack of accuracy and precision for quantitative GC/MS analysis due to insufficient cleanup procedures. The advantages of the proposed method rely also on the fact that there is no need for complete chromatographic separation due to the MRM mode. As for the sample preparation according to polymer dissolution, GC-MS/MS provides sensitive, efficient, and reliable results. This method would be a useful tool to control the safety of intravascular administration set and assess unintended harmful substances, especially for the presence of both majority and minority plasticizers at the level of various concentrations.

## 2. Materials and Methods

### 2.1. Chemicals and Reagents

Standard solutions of eight analytes (BBP, DBP, DEHA, DEHP, DNOP, DOTP, DINP, and DIDP; see Table 1 for their full chemical names and information) for GC-MS/MS analysis were purchased from Sigma-Aldrich (St. Louis, MO, USA), and stock solution was at a concentration of 2000 mg/L in hexane. Internal standards of benzyl benzoate (BB) were purchased from Sigma-Aldrich (St. Louis, MO, USA) and used at a concentration of 100 mg/L in hexane as a stock solution. Organic solvents (hexane and THF) of the GC analysis grade were purchased from Burdick & Jackson (Philipsburg, NJ, USA).

### 2.2. Preparation of Samples

50 mg of small pieces from the tubings in medical infusion sets in 5 mL of THF was sonicated at room temperature for 30 min in a glass tube. Subsequently, 10 mL of hexane was added into the glass tube, and the solution was vortexed for the precipitating polymer matrix. After precipitating the polymer matrix, the solution was left as it was for 10 min. 0.5 mL of supernatant extracted samples was transferred into a 2 mL of vial while 100 ng of internal standard was added. A total volume of 1 mL was homogenized and filtered by an Acrodisc 0.2 *µ*m GHP syringe filter.

### 2.3. Analysis Using GC-MS/MS

Analysis was performed by an Agilent 7890B gas chromatograph, equipped with a 7010 mass selective detector triple quadrupole mass spectrometer system (Palo Alto, CA, USA). Chromatographic separation was achieved using a DB-5MS UI (5% diphenyl-95% dimethyl siloxane phase, 30 m × 0.25 mm I. D; 0.25 *µ*m film thickness) from a J&W Scientific (Santa Clara, CA, USA) capillary column. The temperature of injector was 300°C. One microliter of each extract was injected in the split mode (2  :  1). Helium as carrier gas (99.999%) flow was 1 mL/min. The GC oven temperature program was as follows. The initial temperature of 150°C was held for 3 min after injection before it was increased up to 300°C at 10°C/min held for 12 min. Nitrogen (99.999%) was used as collision gas. The running time was 30 min, divided into seven segments of time for each selected product ion to increase sensitivity and selectivity. The transfer line and ion source temperature were set at 250 and 230°C, respectively. The mass spectrometer is tuned on electron impact ionization (EI) at 70 eV in the multiple reaction monitoring (MRM) mode.

## 3. Results and Discussion

### 3.1. Sample Preparation

**Figure 1:**
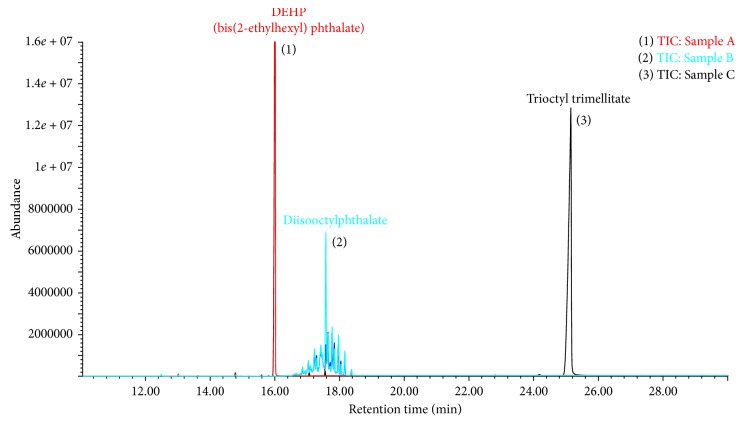
Total ion chromatograms of the hexane extracts after polymer dissolution in tubings of three domestic medical infusion sets. In the TICs, majority plasticizers for each sample are represented in a chromatogram with the three main peaks, each identified by different colors.

Previous studies and conventional extraction techniques, including Soxhlet, solvent extraction, or a method that firstly dissolves the whole polymer and then separates the plasticizers from the PVC by precipitation, are suggested. Due to the large volume of solvent needed and the long procedure times, solvent extraction after the polymer dissolution at room temperature has been used as an alternative efficient and simple technique instead of Soxhlet extraction [10]. In this study, 50 mg of the cutting infusion tube was dissolved in 5 mL of THF and sonicated for 30 min. The mixed solution was left for 10 min or more, and then the polymer was precipitated due to difference in polarity between THF and hexane. According to the solvent used for the phthalate extraction, an organic solvent such as dichloromethane [29] or acetone [30], hexane, and acetonitrile [31] has been used. The selection of different solvents for extraction can have significant effects on the discrimination of target analytes from the sample matrix, as well as the extraction recoveries [32]. Although the advantage of this sample preparation is simple without purification, the matrix effect based on the different organic plastic additives in infusion sets can lead to false results of target analytes [33]. As an extraction solvent after the polymer dissolution, hexane was chosen because the partitioning of the extract with hexane was able to minimize matrix interferences compared with other solvents.  Figure 1 shows the total ion chromatograms of the hexane extracts after polymer dissolution in three domestic infusion sets using a GC-MS system. Sample A clearly showed a large amount of DEHP released from the sample suspected to be the PVC sets. Although the use of DEHP in medical tubing has been restricted, it is still used in medical devices within the medical industrial field. In the case of samples B and C, diisooctylphthalate (DIOP) and trioctyl trimellitate (TOTM) were mainly detected, respectively. These chemicals could be used as alternatives to regulated analytes in domestic medical devices, with their unwanted ions interfering with the detection of the target analytes. For spiking experiments, polyurethane (PU) sample containing none of the eight target analytes was used to evaluate the suitability of the analytical method.

### 3.2. GC-MS Analysis

A gas chromatography coupled with single quadrupole mass spectrometer with selected ion monitoring (SIM) mode has been frequently used to evaluate the analytical performance and validation for quantitative analysis in the clinical research field owing to high sensitivity and the ability to achieve low limits of detection [34]. Based on the test method of the standard operating procedure for the determination of phthalates in PVC products by the U.S. Consumer Product Safety Commission's (CPSC) testing laboratory (LSC) [35], three laboratories were involved in an interlaboratory collaborative study to test the practicability of the modified CPSC method for the quantitative determination of phthalates and assimilated analytes in infusion tubing samples. Accuracy, precision, and linearity as evaluation parameters between laboratories were performed using the sample preparation procedures (Section 2.2) with the same type of commercial GC-MS instrument. All the investigated calibration curves were obtained by the acceptable correlation coefficients (*R*^2^ > 0.99) at five concentrations ranging from 0.05 to 5 *μ*g/g for DBP, BBP, DEHA, DEHP, DNOP, and DOTP and from 0.15 to 15 *μ*g/g for DINP and DIDP, respectively. However, it was found that there was a lack of accuracy and precision from the results of the spike recovery comparison of collaborative studies. The accuracy and precision were assessed by recovery experiments between laboratories using triplicate target analyte free samples spiked with three different concentration points (low, middle, and high) in the calibration range compared with the pure authentic standards. Over 60% of the 144 individual results reported fell outside of the acceptable limits for recoveries (normally ranging from 70 to 130%) and precision (<20% RSD). These results indicated that the analytical method of extraction solvent after the polymer dissolution in the medical infusion sets followed by GC-MS was unsuitable for the determination of trace level concentration of phthalates and assimilated analytes. As mentioned by the U.S. Consumer Product Safety Commission (CPSC), it was intended to address the determination of concentrations of more than 0.1 percentage per plasticized component part of toys for children or child care article in order to protect children from hazard [35]. Therefore, triple quad GC-MS system allows the simplification of sample preparation while maintaining high selectivity and sensitivity when targeting trace level of analytes in complex sample extracts [36].

### 3.3. Optimization of the GC-MS/MS Conditions

**Figure 2:**
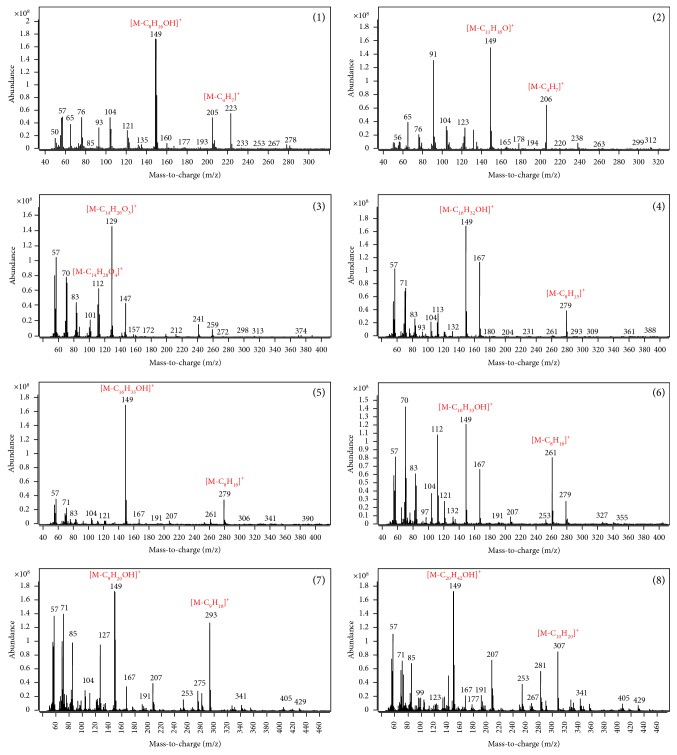
Full-scan MS/MS data of each target analyte to optimized MRM transitions. Each individual parent ion scan experiment is represented as follows: (1) dibutyl phthalate (DBP); (2) benzyl butyl phthalate (BBP); (3) bis(2-ethylhexyl) adipate (DEHA); (4) bis(2-ethylhexyl) phthalate (DEHP); (5) di-n-octyl phthalate (DNOP); (6) dioctyl terephthalate (DOTP); (7) diisononyl phthalate (DINP); (8) diisodecyl phthalate (DIDP).

**Figure 3:**
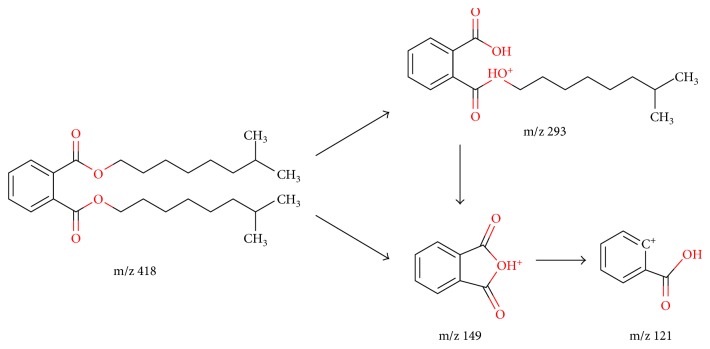
Fragmentation pathway of diisononyl phthalate (DINP) in the MRM mode. A similar fragmentation pattern is found for the other target phthalates. The dominant fragmented ion of m/z 149 in phthalates is able to distinguish among different alternative plasticizers, such as bis(2-ethylhexyl) adipate (DEHA).

For GC-MS/MS analysis using the triple quadrupole, full-scan spectra were obtained to select the precursor ions of each target analyte.  Figure 2 shows the full-scan MS/MS data of each analyte to optimized MRM transitions. A mass-to-charge ratio of 149 or 129 was selected as precursor ions of DBP, BBP, DEHA, and DEHP due to their highest mass intensity. Mass-to-charge ratios of 279, 261, 293, and 307 were selected as precursor ions of DNOP, DOTP, DINP, and DIDP generally considered as confirmation ions for increasing selectivity. Collision-induced dissociation (CID) for selected precursor ions using 99.999% nitrogen was acquired to find the appropriate collision energy of each analyte. To determine collision energies (CEs) for both the quantifying and qualifying MRM ion transitions, they were varied between 2 and 20 eV and optimized as shown in Table 1. This shows that the precursor ions and dominant fragmented ions of phthalates in EI ionization were typically m/z 149, corresponding to the protonated phthalic anhydride ion [C_8_H_5_O_3_]^+^ [37]. The useful dissociation pathway for phthalates was provided by the relatively low collision energy of the molecular ion.  Figure 3 shows the mass spectral fragmentation pathway of DINP in the MRM mode as an example. The molecular ion loses the alkyl fragment accompanied by two hydrogen migrations. Since the molecular ion peak for phthalates with long chain alkyl groups is usually weak, it was not always present in the mass spectra. But the ions loosing alkyl group [M-R]^+^ and those with oxygen [M-OR]^+^ fragments can be a secondary form of identification [38]. In the MRM mode, m/z 293 cleaved again to m/z 149, losing the remaining alkyl fragment as shown in Figure 3. The main product ion from m/z 149 is m/z 121, resulting from fragmentation with loss of the aldehyde group. A similar fragmentation pattern was observed in the other analytes except different alternative plasticizers such as bis(2-ethylhexyl) adipate as shown in Figure 2.

**Figure 4:**
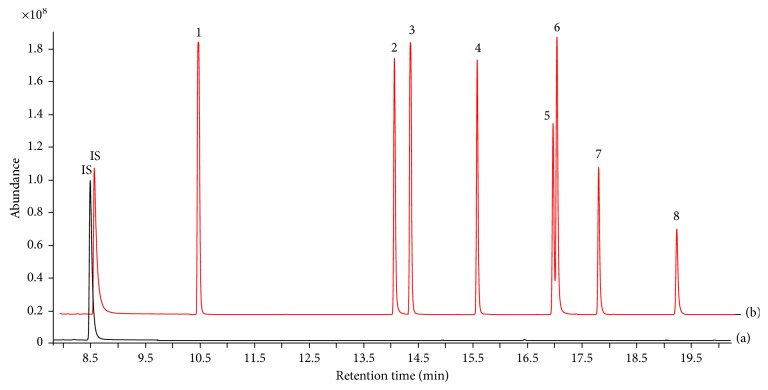
GC-MS/MS MRM chromatograms of matrix blank sample (a) and sample spiked with analytes at 5 *μ*g/g (b). Peak identities are as follows: (1) dibutyl phthalate (DBP); (2) benzyl butyl phthalate (BBP); (3) bis(2-ethylhexyl) adipate (DEHA); (4) bis(2-ethylhexyl) phthalate (DEHP); (5) di-n-octyl phthalate (DNOP); (6) dioctyl terephthalate (DOTP); (7) diisononyl phthalate (DINP); (8) diisodecyl phthalate (DIDP); IS, benzyl benzoate (BB).

To verify the presence of target analytes in matrix blank samples, 5 *μ*g/g (the concentration of solid by weight) was spiked in the matrix sample and the same experimental process was conducted. The spiked and matrix blank sample were analyzed by the same instrument condition.  Figure 4 shows MRM chromatograms of matrix blank sample (a) and those spiked with target analytes (5 *μ*g/g) (b). As a result, target analytes were not observed in the matrix blank sample, and each peak of target analytes were separated clearly in the MRM mode.

### 3.4. Analytical Performance

**Figure 5:**
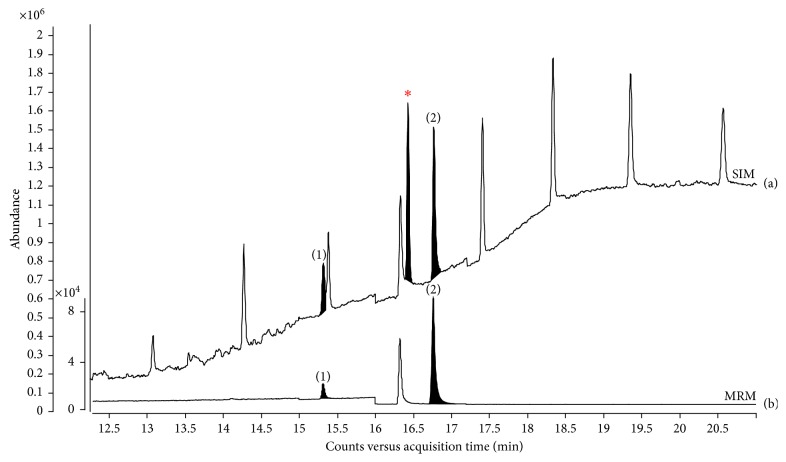
GC-MS SIM chromatogram (a) in comparison with the corresponding GC-MS/MS MRM chromatogram (b) in a tubing of medical infusion set containing bis(2-ethylhexyl) phthalate (DEHP) (1) and dioctyl terephthalate (DOTP) (2). SIM chromatogram shows an inconsistent high noise level and interfering peaks, and false-positive identification of di-n-octyl phthalate (DNOP) is represented by an asterisk in SIM chromatogram, but elimination is clear in MRM chromatogram.

Method validation was performed by spike recovery experiments based on the optimized analytical methods. In this study, benzyl benzoate was used as an internal standard (IS) for target analytes. The calibration curves were generated in the range of 5–500 ng/g using a least-square linear regression analysis. The correlation coefficients were all greater than 0.999 compared with results obtained by GC-MS. The limit of quantification (LOQ) for each analyte was determined in accordance with ICH guidelines [39]. The calculated LOQs ranged from 54.1 to 76.3 ng/g, and they were approximately 20 times lower compared with the results from the latest study which was performed to determine LOQ by the GC/MS method [18]. To validate the accuracy and precision of the analytical method, three replicate analyses of matrix blank samples spiked with the known amounts of the analytes at low and middle concentration levels (17 and 100 ng/g) were prepared and determined by the analytical procedure. The accuracy and precision were assessed by the average recovery and percentage relative standard deviation (%RSD) of three results at each concentration, as shown in Table 2. The average recoveries of eight analytes ranged from 91.8% to 122%, with RSDs ranging from 1.8% to 17.8%. The linearity, LOQ, accuracy, and precision were summarized in Table 2. The developed method was applied to determine the regulated plasticizers in domestic infusion sets. As detected infusion sets containing DEHP (5.4 *μ*g/g) and DOTP (153.7 *μ*g/g), the chromatogram obtained by the GC-MS SIM mode (a) in comparison with the corresponding chromatogram obtained by the GC-MS/MS MRM mode (b) are shown in Figure 5. Due to the presence of relatively high concentrations of DEHP and DOTP, the calibrations with ten times lower concentration of internal standard, but using the same analyte concentration range, were adopted, and their concentrations in the sample were multiplied by the dilution factor. Upon comparing the chromatograms, the superiority of MRM was obvious. In the MRM mode, the baseline noise of the chromatogram was reduced and distinct peaks appeared, whereas the interferences observed in the SIM mode even look like a false-positive DNOP peak. Apparently, MRM gave higher sensitivity and selectivity than the SIM mode for the determination of target analytes in medical infusion sets.

## 4. Conclusion

The suitability of solvent extraction with the polymer dissolution method followed by the GC-MS/MS MRM mode for the determination of eight regulated plasticizers (DBP, BBP, DEHA, DEHP, DNOP, DOTP, DINP, and DIDP) in the tubing of medical infusion sets was described. The validated method was successfully used to analyze the samples of medical infusion sets, maintaining high sensitivity and selectivity. The LOQ of this study using the GC-MS/MS MRM mode was lower than those of other studies using conventional GC-MS. The proposed method could be a useful tool to control the safety of intravascular administration sets and to assess unintended harmful substances, especially plasticizers. Furthermore, it will be helpful for biomonitoring as a methodology for tracking the fate of these chemicals associated with human exposures through direct contact and use because the analytical method enables the trace level determination of target analytes.

## Figures and Tables

**Table 1:** Summary of chemical information and instrument conditions of target analytes.

Compounds	Abbreviation	CAS number	Formula	M.W. (g/mol)	R.T. (min)	Precursor ion	Product ion	Collision energy (eV)
Dibutyl phthalate	DBP	84-74-2	C_16_H_22_O_4_	278.35	10.126	149	121	12
Benzyl butyl phthalate	BBP	85-68-7	C_19_H_20_O_4_	312.37	13.734	149	121	11
Bis(2-ethylhexyl) adipate	DEHA	103-23-1	C_22_H_42_O_4_	370.57	14.032	129	101	2
Bis(2-ethylhexyl) phthalate	DEHP	117-81-7	C_24_H_38_O_4_	390.56	15.251	149	121	13
Di-n-octyl phthalate	DNOP	117-84-0	C_24_H_38_O_4_	390.56	16.642	279	149	3
Dioctyl terephthalate	DOTP	6422-86-2	C_24_H_38_O_4_	390.56	16.716	261	149	8
Diisononyl phthalate	DINP	20548-62-3	C_26_H_42_O_4_	418.61	17.482	293	149	3
Diisodecyl phthalate	DIDP	26761-40-0	C_28_H_46_O_4_	446.67	18.877	307	149	4

**Table 2:** Linearity, LOQ, accuracy, and precision of target analytes in tubings of medical infusion sets.

Compound	*R* ^2^^∗^	LOQ (ng/g)^†^	17 ng/g^‡^		100 ng/g^‡^	
			Accuracy (%)	Precision (%RSD)	Accuracy (%)	Precision (%RSD)
DBP	0.9996	59.5	122	1.8	100	11.4
BBP	0.9996	58.6	104	5.8	103	13.4
DEHA	0.9994	56.9	95.9	4.1	91.8	12.4
DEHP	0.9991	76.3	113	2.0	99.7	13.2
DNOP	0.9993	54.1	116	13.5	98.0	16.0
DOTP	0.9997	75.5	98.8	5.4	118	11.5
DINP	0.9991	72.4	121	17.8	109	9.8
DIDP	0.9994	64.1	109	11.0	92.6	9.2

^∗^The square of the correlation coefficient; ^†^limit of quantitation (LOQ) refers to the lowest concentrations that can be quantified with adequate accuracy and precision; ^‡^the concentrations of the analytes in the spiked samples.
